# Selenium-Binding Protein 1 (SELENBP1) as Biomarker for Adverse Clinical Outcome After Traumatic Spinal Cord Injury

**DOI:** 10.3389/fnins.2021.680240

**Published:** 2021-05-28

**Authors:** Julian Seelig, Raban Arved Heller, Patrick Haubruck, Qian Sun, Georg Jochen Klingenberg, Julian Hackler, Helena Lucia Crowell, Volker Daniel, Arash Moghaddam, Lutz Schomburg, Bahram Biglari

**Affiliations:** ^1^Institute for Experimental Endocrinology, Charité - Universitätsmedizin Berlin, Corporate Member of Freie Universität Berlin, Humboldt-Universität zu Berlin, Berlin Institute of Health, Berlin, Germany; ^2^Heidelberg Trauma Research Group, Department of Trauma and Reconstructive Surgery, Centre for Orthopaedics, Trauma Surgery and Spinal Cord Injury, Heidelberg University Hospital, Heidelberg, Germany; ^3^Department of General Practice and Health Services Research, Heidelberg University Hospital, Heidelberg, Germany; ^4^Raymond Purves Bone and Joint Research Laboratories, Kolling Institute of Medical Research, Institute of Bone and Joint Research, University of Sydney, St Leonards, NSW, Australia; ^5^SIB Swiss Institute of Bioinformatics, University of Zurich, Zurich, Switzerland; ^6^Systems Biology Ph.D. Program, Life Science Zurich Graduate School, ETH Zürich and University of Zurich, Zurich, Switzerland; ^7^Transplantation Immunology, Institute of Immunology, Heidelberg University Hospital, Heidelberg, Germany; ^8^Aschaffenburg Trauma and Orthopaedic Research Group, Centre for Orthopaedics, Trauma Surgery and Sports Medicine, Hospital Aschaffenburg-Alzenau, Aschaffenburg, Germany; ^9^Department of Paraplegiology, BG Trauma Centre Ludwigshafen, Ludwigshafen, Germany

**Keywords:** diagnostic biomarker, *in vitro* diagnostic test, trace element, neuroregeneration, neurotrauma

## Abstract

**Introduction:** Traumatic spinal cord injury (TSCI) presents a diagnostic challenge as it may have dramatic consequences for the affected patient. Additional biomarkers are needed for improved care and personalized therapy.

**Objective:** Serum selenium binding protein 1 (SELENBP1) has been detected in myocardial infarction, reflecting hypoxic tissue damage and recovery odds. As SELENBP1 is usually not detected in the serum of healthy subjects, we tested the hypothesis that it may become detectable in TSCI and indicate tissue damage and regeneration odds.

**Methods:** In this prospective observational study, patients with comparable injuries were allocated to three groups; vertebral body fractures without neurological impairment (control “C”), TSCI without remission (“G0”), and TSCI with signs of remission (“G1”). Consecutive serum samples were available from different time points and analyzed for SELENBP1 by sandwich immunoassay, for trace elements by X-ray fluorescence and for cytokines by multiplex immunoassays.

**Results:** Serum SELENBP1 was elevated at admission in relation to the degree of neurological impairment [graded as A, B, C, or D according to the American Spinal Injury Association (AISA) impairment scale (AIS)]. Patients with the most severe neurological impairment (classified as AIS A) exhibited the highest SELENBP1 concentrations (*p* = 0.011). During the first 3 days, SELENBP1 levels differed between G0 and G1 (*p* = 0.019), and dynamics of SELENBP1 correlated to monocyte chemoattractant protein 1, chemokine ligand 3 and zinc concentrations.

**Conclusion:** Circulating SELENBP1 concentrations are related to the degree of neurological impairment in TSCI and provide remission odds information. The tight correlation of SELENBP1 with CCL2 levels provides a novel link between Se metabolism and immune cell activation, with potential relevance for neurological damage and regeneration processes, respectively.

## Graphical Abstract

The pathophysiology of traumatic spinal cord injury (TSCI) can be divided into two major phases. **(A)** The mechanical trauma is followed within minutes by a secondary phase consisting of local complex and intertwined acute responses, intercellular signaling and cell activity regulating pathways. Inflammatory processes, oxidative stress and hypoxia, leading to cell damage and death, and specific cell contents are released into the circulation **(B)**. The motor and sensory deficits upon TSCI are assessed by using the American Spinal Injury Association (ASIA) impairment scale (AIS), ranging from AIS A as a complete absence of any motor and sensory functions under the lesion site, to AIS E with complete preservation of motor and sensory functions. **(C)** The concentrations of serum SELENBP1 were elevated in patients classified as AIS A as compared to less severely affected patients classified as AIS B, C or D. A cut-off was deduced [(SELENBP1) > 30.2 μg/L], reliably predicting whether a patient belongs to the group showing neurological recovery (G1) or not (G0) within 3 months after the trauma. The figure was created by using https://biorender.com.

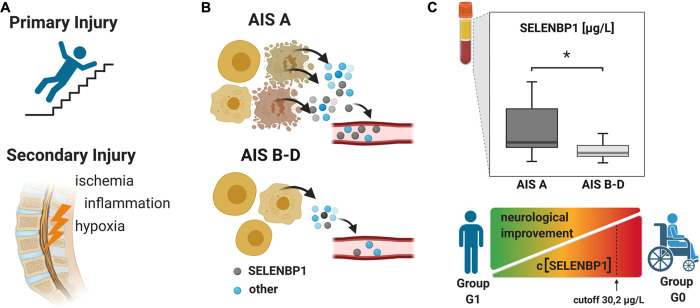

## Introduction

Traumatic spinal cord injury (TSCI) remains one of the most severe injuries and affects predominantly young patients (Furlan et al., 2013; Spinal Cord Injury [SCI], 2016). On a pathophysiological level, the primary injury phase is characterized mainly by the mechanical disruption of the spinal cord (SC) due to shearing, laceration, acute stretching, and sudden acceleration-deceleration events (Baptiste and Fehlings, 2006; Rowland et al., 2008). Hereafter, a secondary injury phase is driven by complex inflammatory responses, involving excitotoxicity, ischemia/hypoxia, inflammation, increased spinal cord intraparenchymal pressure, and oxidative stress. Ultimately, these processes determine the extent of neuronal loss after the mechanical insult (Kwon et al., 2004; Shadgan et al., 2019). Finally, the chronic phase is characterized by adaptive processes, recovery, or autonomic dysregulations (Kwon et al., 2004; Norenberg et al., 2004; Rowland et al., 2008; Moghaddam et al., 2015). Due to the highly dynamic nature of these processes, an informative assessment of the remaining or regained neurological functions after TSCI can only be conducted after months, when a new balance is established. Objective and early biomarkers for the extent of damage with potential relevance for remission and prognosis are urgently needed (Kwon et al., 2019).

The essential trace elements selenium (Se), copper (Cu), and zinc (Zn) are of crucial relevance for immune responses and neurological repair processes (Levenson, 2005; Ma et al., 2018; Zhang et al., 2020) partly due to trace element containing proteins with enzymatic or transport functions. Selenoprotein P (SELENOP) is a circulating Se transport protein with peroxidase activity, ceruloplasmin (CP) is an oxidoreductase and Cu transporter, and the intracellular Cu/Zn superoxide dismutase is an essential component of the antioxidative defense (Besold et al., 2016; Kielczykowska et al., 2018; Lewandowski et al., 2019). Thus, trace elements, when available to the organism in physiological concentrations, exert a beneficial influence on the regulation of various immune cells (Avery and Hoffmann, 2018), are facilitating in regeneration processes after injuries (Lansdown et al., 2007), or, in the form of SELENOP, influence the survival of neurons exposed to oxidative stress (Yan and Barrett, 1998).

Se-binding protein 1 (SELENBP1) is a poorly characterized parameter of Se metabolism, transport and intracellular accumulation. It can exert enzymatic activity, capable of oxidizing methanethiol (Pol et al., 2018), and it constitutes a potential early biomarker of schizophrenia (Mohammadi et al., 2018). SELENBP1 is located intracellularly under normal conditions, partly in complex with Se-dependent glutathione peroxidase 1 (GPX1) (Diamond, 2015). Its expression is dysregulated in malignant tissue (Hughes et al., 2018; Schott et al., 2018), and it may serve as a biomarker of adipocyte differentiation (Steinbrenner et al., 2019). There are indications that the protein contributes to redox control, affecting cell differentiation and motility (Elhodaky and Diamond, 2018). Moreover, extracellular SELENBP1 can be detected in blood following myocardial infarction or during cardiac surgery, where serum SELENBP1 levels correlate to tissue damage and hypoxic stress (Kühn et al., 2019; Kuhn-Heid et al., 2019).

Based on these findings, we hypothesized that TSCI might be associated with an increase in circulating SELENBP1 concentrations and that elevated serum SELENBP1 at an early stage after injury may correlate to the severity and the neurological outcome of this devastating condition. Accordingly, the aim of this study was to determine circulating SELENBP1 concentrations and to analyze whether this parameter correlates to the extent of neurological impairment and clinical outcome after 3 months. In order to facilitate the evaluation of the analyses and to identify potential associations with other potentially relevant parameters, circulating chemokines, trace elements and associated biomarkers were analyzed in parallel.

## Materials and Methods

### Study Design

This clinical prospective observational study has been approved by the local ethics committee of the University of Heidelberg (S514/2011). It was registered (Study-ID: DRKS00009917/ Date of Registration: 23.03.2016/Universal Trial Number (UTN): U1111-1179-1620) at the German Clinical Trial Register (Deutsches Register Klinischer Studien—DRKS). Data collection and processing were performed according to good scientific practice, and the manuscript was composed according to the STROBE statement (von Elm et al., 2008). All study participants signed an informed consent form and agreed to participate. The patients were informed that they could choose to leave the study without reason at any time and that this decision will not affect further treatment in any way.

### Source of Clinical Data

**FIGURE 1:**
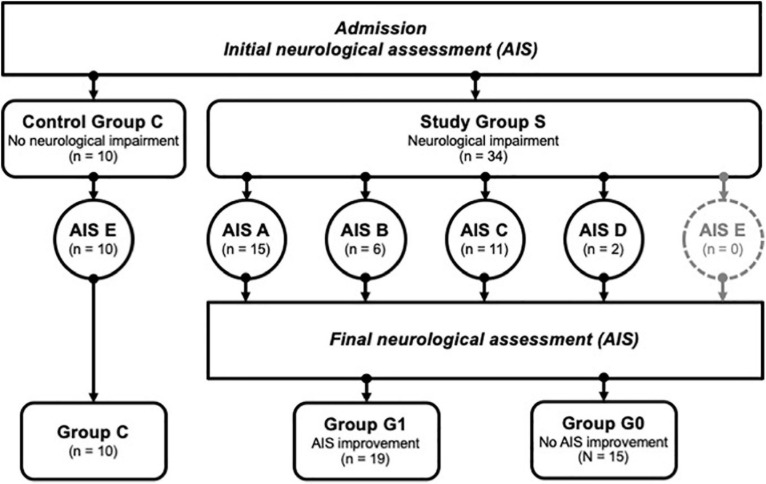
Patient identification and allocation scheme. A set of *n* = 44 patients were successfully enrolled into the study, and divided into groups with no neurological impairment (Control Group C) vs. patients with neurological impairment (Study Group S). The latter group was subdivided further according to the severity of the symptoms using the AIS categories A-D, where A relates to the most severe condition. AIS improvement was assessed 3 months after enrolment, allowing a subdivision of S into group G1 (improvement) or G0 (no improvement). Group C; controls, group G1; patients with neurological remission, group G0; patients without neurological remission, AIS; American Spinal Injury Association (ASIA) Impairment Scale.

The clinical data were collected during the examinations and consecutively provided by the hospital database. Inclusion criteria were defined as the occurrence of at least one fracture of the spine with accompanying sensorimotor deficits resulting from TSCI. Fractures were classified according to the AO classification (Magerl et al., 1994) and the occurrence of sensorimotor deficits described as neurological level of injury (NLI). The NLI is defined as the lowest neurological level, where both motor and sensory functions are intact. Exclusion criteria were non-traumatic spinal cord injury (SCI), traumatic brain injury, severe abdominal trauma, traumatic amputation of extremities, coma, or any additional life-threatening trauma apart from the SCI (Heller et al., 2020). During the study period, no methylprednisolone sodium succinate was provided to the participating patients. The patients included in the study were grouped into the study group S (*n* = 34), which was retrospectively divided into two subgroups G0 and G1 according to the clinical outcome after 3 months. G1 (*n* = 19) included patients with neurological remission, and group G0 (*n* = 15) consisted of patients without any improvement of the neurological functions within 3 months after injury. Ten subjects with vertebral fractures without neurological impairment were analyzed and served as the control group C (*n* = 10). The detailed patient allocation to the groups is visualized (Figure 1), and patient characteristics are provided (Tables 1, 2).

**TABLE 1:** Descriptive depiction of patient characteristics of subjects in the study group S and the control group C.

**[A]**	**Study Group S**	**[B]**	**Control Group**
	**(*N* = 34)**		**C (*N* = 10)**
**Age**		**Age**	
Median	41.0	Median	41.0
(IQR)	(15.0, 77.0)	(IQR)	(27.0, 71.0)
**Sex**		**Sex**	
Female	8 (23.5%)	Female	5 (50.0%)
MALE	26 (76.5%)	Male	5 (50.0%)
**AIS initial**		**Etiology**	
A	15 (44.1%)	Fall	7 (70.0%)
B	6 (17.6%)	Traffic	3 (30.0%)
C	11 (32.4%)		
D	2 (5.9%)	**AO**	
		A	7 (70.0%)
**AIS final**		B	3 (30.0%)
A	11 (32.4%)		
B	3 (8.8%)		
C	6 (17.6%)		
D	14 (41.2%)		
**Etiology**			
Fall	20 (58.8%		
Other	3 (8.8%)		
Traffic	11 (32.4%)		
**NLI**			
Cervical	13 (38.2%)		
Lumbar	8 (23.5%)		
Thoracic	13 (38.2%)		
AO*			
A	20 (60.6%)		
B	5 (15.2%)		
C	8 (24.2%)		

NLI, Neurological Level of Injury; AO, AO-Classification; AIS, ASIA (American Spinal Injury Association) Impairment Scale. Age is expressed as median years with their corresponding IQR. *One patient in the subgroup G0 suffered an isolated contusion of the spinal cord without vertebral fracture, so no AO classification was assessed.

**TABLE 2:** Clinical characteristics of subjects in the groups G0 (*n* = 15) and G1 (*n* = 19), and subgroups AIS A (*n* = 15) and AIS B-D (*n* = 19).

	**G0 (*n* = 15)**	**G1 (*n* = 19)**	***p*-value**	**AIS A (*n* = 15)**	**AIS B-D (*n* = 19)**	***p*-value**
Age			0.107			0.238
Median	47.0	32.0		44.0	34.0	
(IQR)	(21.0, 77.0)	(15.0, 75.0)		(21.0, 75.0)	(15.0, 77.0)	
Sex			1.000			1.000
Female	3 (20.0%)	5 (26.3%)		4 (26.7%)	4 (21.1%)	
Male	12 (80.0%)	14 (73.7%)		11 (73.3%)	15 (78.9%)	
AIS initial			0.001			< 0.001
A	11 (73.3%)	4 (21.1%)		15 (100.0%)	0 (0.0%)	
B	1 (6.7%)	5 (26.3%)		0 (0.0%)	6 (31.6%)	
C	1 (6.7%)	10 (52.6%)		0 (0.0%)	11 (57.9%)	
D	2 (13.3%)	0 (0.0%)		0 (0.0%)	2 (10.5%)	
AIS final			< 0.001			< 0.001
A	11 (73.3%)	0 (0.0%)		11 (73.3%)	0 (0.0%)	
B	1 (6.7%)	2 (10.5%)		2 (13.3%)	1 (5.3%)	
C	1 (6.7%)	5 (26.3%)		2 (13.3%)	4 (21.1%)	
D	2 (13.3%)	12 (63.2%)		0 (0.0%)	14 (73.7%)	
Etiology			0.588			0.588
Fall	9 (60.0%)	11 (57.9%)		9 (60.0%)	11 (57.9%)	
Other	2 (13.3%)	1 (5.3%)		2 (13.3%)	1 (5.3%)	
Traffic	4 (26.7%)	7 (36.8%)		4 (26.7%)	7 (36.8%)	
NLI			0.083			0.083
Cervical	4 (26.7%)	9 (47.4%)		4 (26.7%)	9 (47.4%)	
Lumbar	2 (13.3%)	6 (31.6%)		2 (13.3%)	6 (31.6%)	
Thoracic	9 (60.0%)	4 (21.1%)		9 (60.0%)	4 (21.1%)	
AO*			0.039			0.015
A	5 (35.7%)	15 (78.9%)		6 (40.0%)	14 (77.8%)	
B	4 (28.6%)	1 (5.3%)		5 (33.3%)	0 (0.0%)	
C	5 (35.7%)	3 (15.8%)		4 (26.7%)	4 (22.2%)	

The Kruskal-Wallis test for comparison of two independent samples and the Fisher’s exact test were used to assess the differences in numeric and categorical variables. NLI, Neurological Level of Injury; AO, AO-Classification; AIS, ASIA (American Spinal Injury Association) Impairment Scale. Age is expressed as median years with their corresponding IQR. Neurological remission was defined as improvement in AIS within 3 months after the trauma. *One patient in the subgroup G0 suffered an isolated contusion of the lumbar spinal cord without vertebral fracture, so no AO classification was assessed.

### Source of Material

**FIGURE 2:**
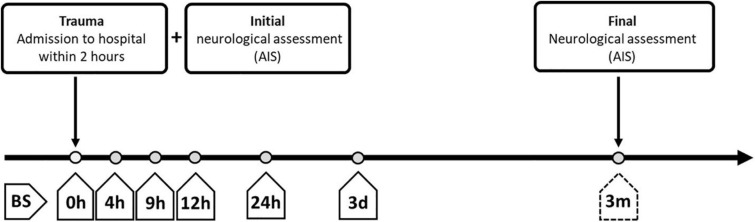
Overview of standardized blood sampling and neurological assessment protocols for patients with severe traumatic injuries. The study enrolled patients who were administered to the hospital within 2 h after the traumatic injury. Blood samples (BS) were collected at different time points after admission, starting immediately at entry into the hospital (0 h after injury; BS 0 h) and extending until the time of final neurological assessment at 3 months after admission (BS 3 m). BS, blood sample; AIS, American Spinal Injury Association (ASIA) Impairment Scale; h, hours; d, days; m, months.

Venous blood samples were collected in the Department of Paraplegiology at the BG Trauma Center Ludwigshafen from TSCI patients from 2011 to 2018. Consecutive blood samples were drawn from patients at specific time points covering the period from the time of admission until 3 months after injury according to our study protocol (Figure 2). All blood samples were treated routinely according to the same standard procedure; 20 min of coagulation at room temperature, centrifugation at 3,000 rpm with an RCF of 1,000 g, aliquoting into sterile tubes and storing at −80°C until analysis or transport on dry ice. Missing samples in the protocol are mostly due to urgent interventions. The laboratory analyses for SELENBP1 and cytokine concentrations were conducted by staff blinded to patient identities and clinical data in the Institute for Experimental Endocrinology of the Charité–Universitätsmedizin Berlin and the Institute of Immunology at Heidelberg University Hospital, respectively.

### Sample Analysis

#### Trace Element Analyses

Trace element concentrations were determined by total reflection X-ray fluorescence (TXRF) analysis, essentially as described (Hughes et al., 2016; Heller et al., 2019). Briefly, serum samples were diluted with a Gallium standard and applied to polished quartz glass discs. After drying, a benchtop TXRF device (PicoFox S2, Bruker Nano, Berlin, Germany) was used for recording the fluorescence spectra emitted from the elements upon X-ray excitation. An internal laboratory quality control was included in each measurement run, and all samples were measured in duplicate (Heller et al., 2020). The inter-assay coefficient of variation (CV) was below 10%, as determined with a commercial standard serum (Sero AS, Seronorm, Billingstad, Norway) (Hughes et al., 2016; Heller et al., 2019).

#### Ceruloplasmin (CP) and Selenoprotein P (SELENOP) Quantification by Sandwich Immunoassays

Serum samples were tested for SELENOP and CP concentrations. To this end, a validated enzyme-linked immunosorbent sandwich assay specific for human SELENOP (selenOtest^TM^, ELISA) (Hybsier et al., 2017) was used according to the manufacturer’s instructions (selenOmed GmbH, Berlin, Germany). Serum CP concentrations were determined by a sandwich ELISA using a pair of specific monoclonal antibodies in combination with a commercial human CP standard (CP, catalog number 187-51, Lee BioSolutions, Maryland Heights, MO 63043, United States) as described earlier (Hackler et al., 2020).

#### Selenium Binding Protein 1 (SELENBP1) Quantification by LIA

Serum SELENBP1 concentrations were analyzed by a recently established luminometric immunoassay (LIA) (Kühn et al., 2019). Quality of measurements was verified by including two human serum standards in each assay run. Intra- and inter-assay variations of SELENBP1 concentrations were below 15% during the analyses.

#### Cytokine Quantification via Multiplex Bead-Based Immunoassays

Multiplex bead-based immunoassays were used to quantify a set of human chemokines and cytokines (Luminex Performance Human High-Sensitivity Cytokine Panels). Serum concentrations of CCL-2, CCL-4, MMP-2, MMP-8, IL-8, and IL-10 were assessed. The determination was performed according to the manufacturer’s instructions (R&D Systems, Minneapolis, MN, United States).

### Outcome

The American Spinal Injury Association (ASIA) impairment scale (AIS) was used to describe the functional impairment in TSCI patients. The neurological functions were graded as A, B, C, or D by experienced examiners applying the International Standards for Neurological Classification of SCI (ISNCSCI). To quantify the neurological deficit according to the AIS various parameters such as sensitivity, motor function, muscle strength, and the level of the paraplegia are considered. Hence, AIS A grade represents the complete loss of all motor and sensory functions below the site of injury. Whereas AIS B-D constitute incomplete deficits with remaining sensory and/or motor qualities, with B containing the least and D the most functions. Physiological findings without neurologic impairment are classified as AIS E (Table 3). Initial examinations (AIS initial) were performed within 72 h after admission in awake and responsive patients, and final examinations (AIS final) took place at 3 months after the trauma (Burns and Ditunno, 2001). Neurological remission was defined as an improvement of AIS grades within 3 months after the trauma. The initial AIS is illustrated in Figure 1 and Tables 1, 2.

**TABLE 3:** The American Spinal Injury Association Impairment Scale (AIS).

**AIS grade**		**Clinical state**
A	Complete	No motor or sensory function is preserved in the sacral segments S4-S5
B	Incomplete	Sensory but not motor function is preserved below the NLI and includes the sacral segments S4-S5
C	Incomplete	Motor function is preserved below the NLI, and more than half of key muscles below the NLI have a muscle grade less than 3
D	Incomplete	Motor function is preserved below the NLI, and at least half of key muscles below the NLI have a muscle grade of 3 or more
E	Normal	Motor and sensory function is normal

*AIS grades from A to E are considering the completeness of paralysis and the motor and sensory function tests.*

### Predictors

The individual protein and trace element concentration patterns were analyzed concerning both the initial AIS and the presence or absence of neurological remission within 3 months after the injury (Table 3).

### Sample Size

Serum samples from this observational study, along with the respective clinical data of the patients have already been analyzed for different parameters in other studies by our research groups. The explorative research studies have been performed with slightly different sets of patient samples, depending on the inclusion criteria combined with the respective availability of a sufficient quantity of serum samples stored in the biobank at the time of analysis. For this reason, the numbers of patients and samples vary across the different analyses, according to availability, volumes, and the specific scientific issue.

### Missing Data

The mean follow-up of available serum samples for analysis within the first 3 days was higher than 75%; missing values were excluded from the pairwise deletion (Kang, 2013).

### Statistical Analysis

Non-parametric test methods were assessed to investigate location shifts between groups (Mann-Whitney *U*-test, Kruskal-Wallis test). Categorical variables were evaluated using Fisher’s exact test.

As this is an exploratory *post-hoc* analysis, all *p*-values are to be interpreted descriptively, and no adjustment for multiple testing was adopted. The statistical tests are using an α-level of 0.05, and statistical significance was defined as *p* > 0.05 (n.s.), *p* < 0.05 (^∗^), *p* < 0.01 (^∗∗^), or *p* < 0.001 (^∗∗∗^). For SELENBP1 at admission, an optimal cut-off for the differentiation between G0 and G1 was estimated based on the Odds Ratio (OR). All statistical calculations were performed with R version 4.0.2 (R Development Core Team, 2015). Figures were created by using the package “ggplot2” (Wickham, 2009).

## Results

A total of 44 patients were eligible for the current study, including 34 patients with neurological impairment (study group S), divided into 19 patients with remission (group G1) and 15 patients without remission (group G0). The other 10 subjects with vertebral fracture without neurological impairment (group C) served as a control group (Figure 1).

### Patients

Out of all 34 patients in the study group S (G0+G1), eight were female, and 26 were male with an average age of 41 years (IQR 15, 77 years). The TSCI was caused by a fall in 59% of cases, and by accident in 32% of cases. The injuries in the control group (including five males and five females with an average age of 41 years) resulted from a fall in about 70% of cases or from an accident in the remaining 30% of cases. Within the study group S, there were no significant differences regarding age, sex, etiology, NLI or AO classification between the patients with and without neurological remission. The distribution of AIS grades between the groups G0 and G1 differed significantly both at admission (*p* = 0.001) and at discharge (*p* < 0.001). An overview of the patients’ characteristics is shown in Tables 1, 2.

### Biochemical Analysis of the Serum Samples

**FIGURE 3:**
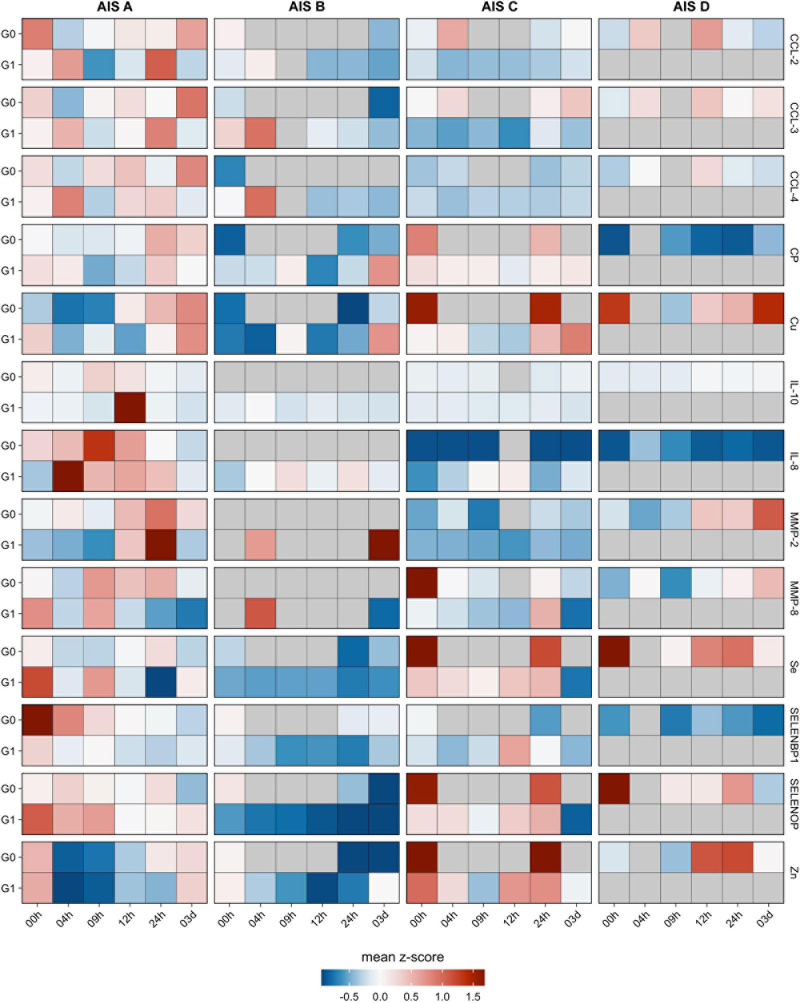
Comparison of cytokines, metalloproteases, trace elements and related biomarkers in relation to neurological remission. The cytokines CCL-2, CCL-3, CCL-4 along with the metalloproteases MMP-2 and MMP-8 were analyzed from the serum samples of patients with TSCI in relation to remission (G1) or no remission (G0). In addition, the trace elements Cu, Fe, Se, and Zn as well as the Se-binding proteins SELENBP1 and SELENOP along with the Cu transporter CP were quantified in parallel. The heat maps indicate the relative concentration differences of these serum parameters in the two groups of TSCI patients (G0 and G1) in relation to the control group C. In addition, relative concentration differences are depicted in regard to the clinical severity of the neurological deficit, classified as AIS A–D according to the American Spinal Injury Association (AISA) impairment scale (AIS). Mean z-scores are indicated as color code.

The analysis of the serum samples indicated that Se, Zn, Cu, SELENOP, SELENBP1, CP, CCL-2, CCL-3, MMP-8, MMP-10, IL-8, and IL-10 were detectable in measurable concentrations. Most of the parameters analyzed displayed impairment-dependent concentration differences according to AIS grades A vs. B-D early in the post-injury period and differed in relation to injury when comparing the groups G0 and G1 vs. C (Figure 3).

### Major Findings

#### Serum SELENBP1 Concentrations Are Elevated With the Severity of Impairment

**FIGURE 4:**
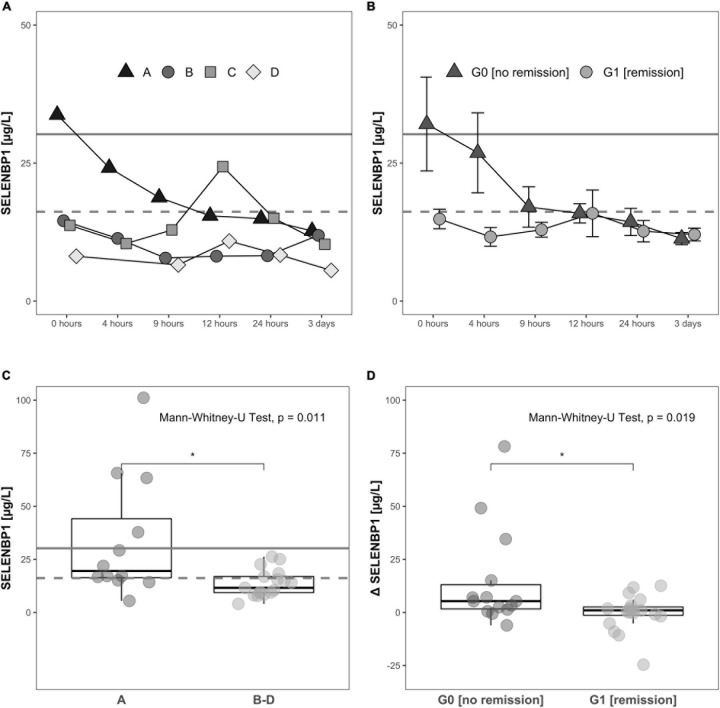
Relation of serum SELENBP1 to the functional impairment at admission and neurological remission. Differences in SELENBP1 concentrations are shown as time-resolved line plots **(A,B)** or box plots **(C,D)**. The mean SELENBP1 levels (16.2 μg/L) of the control group C are depicted as dashed line. Changes (Δ SELENBP1) were calculated by subtracting the late (12 h–3 day) from the early (0–12 h) SELENBP1 concentrations **(D)**. Serum SELENBP1 at hospital admission (0 h) was elevated in patients classified as A by the AIS scheme (most severe) in comparison to patients with a **(B–D)** rating (less severe) **(A,C)**. Similarly, serum SELENBP1 is particularly elevated during the first 3 days after injury in patients not undergoing remission **(B,D)**. The calculated cut-off (30.2 μg/L) for the SELENBP1 threshold as a prognostic marker for remission is indicated as solid line. Values are expressed as mean ± SEM. The Mann-Whitney-U-Test assessed significant differences between two groups; *p < 0.05.

Increased SELENBP1 concentrations were detected especially at early time points available for analysis, i.e., directly at admission to hospital (0 h). The patients with severe injury and an AIS classification of A exhibited relatively high concentrations of SELENBP1 as compared to the other patients, suggesting a relation of acute serum SELENBP1 elevations to the severity of neurological impairment (Figure 4A).

#### Dynamic Changes in Circulating SELENBP1 Are Related to Clinical Outcome

The SELENBP1 concentrations during the first 3 days in relation to the clinical outcome as assessed 3 months after injury differed significantly between the two groups of subjects with TSCI. Patients without remission in group G0 showed relatively high SELENBP1 concentrations at admission (0 h), as compared to the patients with remission in group G1, who had low concentrations throughout the full observation period. The elevated SELENBP1 concentrations in G0 decreased steadily within the first 9 h, while the low SELENBP1 levels in G1 remained constant (Figure 4B).

A direct comparison of serum SELENBP1 concentrations at the time of admission (0 h) highlights the significantly elevated SELENBP1 levels in relation to the extent of the neurological impairment after TSCI, i.e., the patients with most severe injury classified by the AIS system as A displayed highest SELENBP1 (Figure 4C). The Mann-Whitney test indicated that when comparing the initial three samples with the later time points (0 h, 4 h, 9 h vs. 12 h, 24 h, 72 h) the dynamic decrease in SELENBP1 concentrations (Δ SELENBP1) was significantly greater for in G0 (no remission) (Median = 5.32) than G1 (remission) (Median = 0.99), *W* = 197, *p* = 0.019 (Figure 4D). Based on the data obtained for serum SELENBP1 concerning remission, a cut-off of 30.2 μg/L was calculated for allocating patients either to G0 or to G1, providing 98.7% sensitivity, specificity of 12.3%, an accuracy of 58.6%, and an odds ratio of 10.4. This diagnostic cut-off is indicated as a solid line, whereas the SELENBP1 level of controls is indicated as a dashed line at 16.2 μg/L (Figures 4A–C).

#### Correlation Analysis of SELENBP1 With Parameters of Se Status and Covariates in TSCI

**FIGURE 5:**
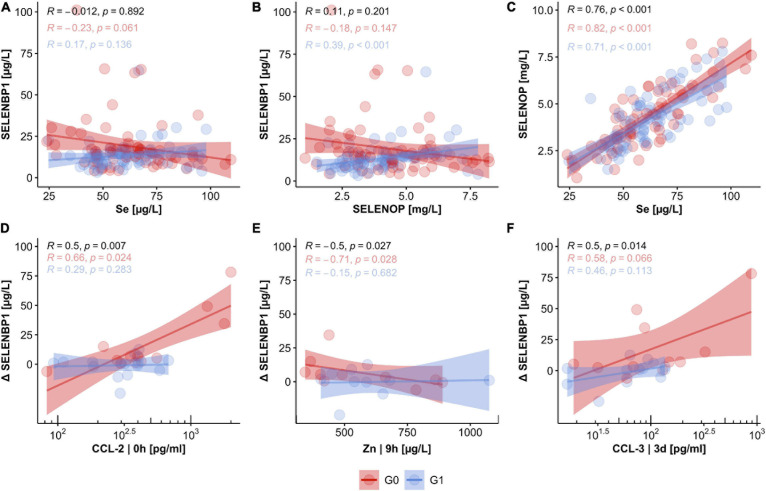
Relation of circulating SELENBP1 concentrations to established biomarkers of Se status and potential covariates in TSCI. The concentrations of circulating SELENBP1 were not associated with either serum Se levels **(A)** or SELENOP concentrations **(B)**, whereas a tight linear correlation between total serum Se and SELENOP concentrations was given **(C)**. Concentrations of CCL-2, Zn, and CCL-3 were strongly associated with declining serum SELENBP1 in patients without neurological improvement after 3 months. In contrast, no significant coherence of CCL-2, Zn, and CCL-3 concentrations with SELENBP1 were observed in the group of patients with neurological improvement in G1 **(D–F)**. Correlation analysis by Pearson, R indicates the correlation coefficient, and p indicates significance, provided on top of the graphics for all samples (black), and the two groups of patients separately (G0; red, G1; blue).

SELENBP1 concentrations were not significantly related to the other Se status biomarkers. The interrelations were characterized by low correlation coefficients of *R* = −0.012 for SELENBP1 with total serum Se (Figure 5A), and *R* = 0.110 for SELENBP1 with SELENOP, respectively (Figure 5B). Concerning the outcome of neurological remission, the correlations of SELENBP1 with Se or SELENBP1 with SELENOP tended to point into opposite directions for patients in group G0 vs. G1 (Figures 5A,B). These findings suggest that serum SELENBP1 is not a surrogate marker of blood Se status in the patients. As expected, total serum Se and SELENOP showed the typically strong and linear interrelation with a correlation coefficient of *R* = 0.76 in the samples from patients with TSCI, irrespective of final remission (Figure 5C). Next, correlations between SELENBP1 concentrations and additional trace elements and cytokines were analyzed to identify other potential covariates. Three parameters correlated significantly with the SELENBP1 decline (Δ SELENBP1; delta 3 day–0 h), i.e., CCl-2 at 0 h (*p* = 0.007, Figure 5D), Zn at 9 h (*p* = 0.027, Figure 5E) and CCL-3 at 3 day (*p* = 0.014, Figure 5F). Concerning the clinical outcome 3 months after TSCI in the group of non-improving patients (G0), strong positive correlations of Δ SELENBP1 were observed for CCL-2 (*R* = 0.66) and CCL-3 (*R* = 0.58), and a negative correlation for Zn (*R* = −0.71). In contrast to these correlations, only moderate and non-significant interrelations were observed in the group of recovering patients in G1 (Figures 5D–F).

#### Relation of Serum SELENBP1 Levels to Trace Elements and Cytokines

An explorative correlation analysis was conducted between the SELENBP1 concentrations with the analyzed trace element parameters and cytokine concentrations. A strong linear association between the initial SELENBP1 concentrations and the dynamics of SELENBP1 decline over the initial 3-day study period was observed (0 h; *R* = 0.94, 4 h; *R* = 0.86, 9 h; *R* = 0.58). To assess differences in all parameters between the groups G0 and G1 with respect to the initial AIS scores, the corresponding log-fold changes (logFC) for each point in time in G1/G0 were calculated. The analysis indicated a negative relationship between SELENBP1 and the group of chemotactic ligands CCL-2, CCL-3, and CCL-4. This interaction was most substantial when comparing the initial concentrations of the most severely impaired patients (AIS group A) in the group with remission (G1) vs. the group with no remission (G0) (Supplementary Figure 1).

## Discussion

The fundamental need for novel therapies improving the neurological recovery of patients who suffer from SCI remains an urgent research issue. However, specific treatments that appeared promising in a pre-clinical setting regrettably failed to show beneficial effects in clinical SCI trials (Tator, 2006). A reason for the controversial study results may be found in the underlying pathophysiological and biochemical processes that are setting in upon SCI and which may fundamentally differ in extent, dynamics and interrelation between the animal models used and the acutely injured human subjects (Kwon et al., 2015). This challenge is most difficult to address, given the paucity of molecular data on the intracellular signaling events, metabolic responses to trauma in patients and the lack of informative diagnostic biomarkers. In addition, the available instrumentation for estimating the extent of injury and predicting the outcome after a TSCI is limited, and reliability of both diagnosis and prognosis mainly depends on the experience and knowledge of the particular clinical examiner. In order to gain further insights and test candidate biomarkers, we have standardized some of the essential parameters and introduced a transparent blood sampling, clinical assessment and laboratory analysis scheme.

Our results suggest that the analysis of serum SELENBP1 may contribute to an improved initial clinical assessment after TSCI and may provide valuable insights into the pathophysiology and individual prognosis. Significant differences in SELENBP1 were detected with regards to degree of neurological impairment (severe AIS A vs. AIS B-D), and with respect to the clinical outcome after 3 months (remission vs. no remission). The patients who displayed elevated serum SELENBP1 concentrations at admission (above the cut-off at 30.2 μg/L) presented with the most severe impairment (classified as AIS A) and were most unlikely to achieve neurological remission. The other severely impaired patients with the same classification of AIS A, but with SELENBP1 below this threshold showed a high chance for recovery. Using this SELENBP1 threshold, a prediction for remission was enabled with a sensitivity of 98.7% and an odds ratio of 10.4, i.e., with a diagnostically valuable and acceptable degree of reliability.

Due to the observational nature of this study, causal interrelationships cannot be deduced. However, some knowledge of SELENBP1 is available from prior studies. SELENBP1 constitutes a highly conserved protein between species, that may be critical for specific physiological functions, potentially including cell differentiation, protein degradation, intra-Golgi vesicular transport, cell motility and redox modulation (Elhodaky and Diamond, 2018). Its expression is strongly affected by hypoxia, as shown in the context of cancer (Huang et al., 2012; Jeong et al., 2014) and cardiovascular research (Kühn et al., 2019; Kuhn-Heid et al., 2019). It would be highly interesting to study whether serum SELENBP1 is related to locally depressed oxygen levels, employing suitable monitoring techniques such as near-infrared spectroscopy (Casha and Christie, 2011; Ryken et al., 2013; Hawryluk et al., 2015). The assumption that increased SELENBP1 is related to hypoxia and cell death resulting from increased ischemia in TSCI is further supported by the positive correlation with CCL-2 (*R* = 0.66), that is known as a hypoxia-responsive cytokine (Mojsilovic-Petrovic et al., 2007). It is also consistent with our prior study (Heller et al., 2017), where patients with no improvement in neurological function initially showed increased CCL-2 levels, with a resulting induction of monocyte migration, monocyte proliferation and differentiation (Kiguchi et al., 2010).

Previous studies indicated that peripheral trace element dynamics and concentration changes in the trace element biomarkers are associated with the clinical outcome after TSCI (Heller et al., 2019, 2020; Sperl et al., 2019; Seelig et al., 2020). It was thus hypothesized that there might be a close correlation between serum Se, SELENOP and SELENBP1 concentrations. Unexpectedly, no significant interrelation between SELENBP1 and the other Se status biomarkers was observed, neither in the group with non-remission nor in the remission group. This result highlights that SELENBP1 may not directly affect extracellular serum Se status, potentially due to its relatively low serum concentrations and the different origins of these proteins (mainly liver in case of SELENOP, kidney in case of GPX3 vs. damaged tissue in case of SELENBP1).

Due to the divergent degrees of injuries within AIS classes from A to E, SELENBP1 concentration dynamics may provide a direct insight into the individual burden of hypoxic stress on a cellular level. Combined with additional diagnostic parameters, SELENBP1 monitoring might pave the way for a more detailed and quantitative clinical assessment strategy after TSCI, thereby supporting the established INSCCI examinations.

Future studies are required to test for a correlation of SELENBP1 dynamics with intraspinal pressure (Phang et al., 2015; Chen et al., 2018; Saadoun and Papadopoulos, 2020), and local tissue oxygenation at the injury site (Kurita et al., 2020). In addition to the sensor-derived data, SELENBP1 might also provide information about other tissues that are damaged or at-risk for degeneration, and remote from the sensor. The SELENBP1 concentrations may also reflect other sorts of injury, including micro-bleedings that might not be detectable by current imaging techniques such as MRI or CT scan. The relevance of these processes and their contribution to the global burden of injured neural tissue and the neuroinflammatory signaling in the second phase after TSCI still need to be evaluated. Recent findings support the importance of spinal cord perfusion pressure (SCPP) monitoring with respect to metabolic characteristics of the injured tissue concerning the chances of neurological remission after TSCI. The data indicate a close correlation between the individual SCPP and metabolic profiles at the injury site, estimated via tissue glucose, lactate, pyruvate, glutamate and glycerol by surface microdialysis (Saadoun and Papadopoulos, 2020). This information might support the identification of individuals with lower potential for remission, and aid in personalized therapy.

With a better characterization of the regulation and function of SELENBP1 in these tissues, its potential role in diseases such as TSCI may be better understood, and SELENBP1 may become a novel and valuable biomarker for diagnostic, monitoring, and prognostic purposes in acute and potentially also in chronic injuries.

### Limitations

Despite the relevant and convincingly strong interrelations identified, the current study is not free from limitations. The sample size was relatively small, yet it was sufficient to deduce a cut-off for the early detection of patients with a high chance of neurological remission after TSCI by serum SELENBP1. Still, serum trace elements and their protein biomarkers may be surrogate markers, not necessarily affecting disease course directly or reliably reflecting the physiologically relevant intracellular trace element concentrations (Maret and Sandstead, 2006). Furthermore, the data are from an observational study, and are thus not suitable for deducing mechanistic insights. Finally, the pathophysiological and clinical heterogeneity within the AIS groups complicates the interpretation of the results and necessitates an independent verification of this newly identified biomarker in TSCI.

## Conclusion

Our results indicate that the analysis of SELENBP1 concentrations in serum provides promising insights regarding the early assessment of both the injury severity after TSCI in AIS A vs. B, C, or D and the individual chance of neurological remission. Monitoring serum SELENBP1 concentrations could assist clinicians in the initial assessment of patients after TSCI, especially in estimating the remission potential of severely injured patients classified as AIS A. Our results support the notion that SELENBP1 constitutes a promising marker for identifying and assessing cell damage and injury, and this potential should be investigated further in the context of other traumatic or degenerative diseases.

## Data Availability Statement

The raw data supporting the conclusions of this article will be made available by the authors, without undue reservation.

## Ethics Statement

The studies involving human participants were reviewed and approved by the Local Ethics Committee of the University of Heidelberg (S514/2011). Written informed consent to participate in this study was provided by the participants’ legal guardian/next of kin.

## Author Contributions

RH, VD, AM, LS, and BB: conceptualization. JS, RH, QS, JH, LS, and BB: methodology. JS, RH, HC, and LS: software. JS, RH, PH, QS, GK, JH, HC, VD, AM, LS, and BB: visualization and data curation. JS, RH, VD, AM, LS, and BB: validation. JS, RH, HC, VD, AM, LS, and BB: formal analysis. VD, AM, LS, and BB: resources, supervision, and funding acquisition. JS, RH, LS, and BB: writing—original draft preparation. PH, QS, GK, JH, HC, VD, and AM: writing—review and editing. All authors have read and agreed to the published version of the manuscript.

## Conflict of Interest

LS holds shares in selenOmed GmbH, a company involved in Se status assessment and supplementation. The remaining authors declare that the research was conducted in the absence of any commercial or financial relationships that could be construed as a potential conflict of interest.
