# Supplementary material for: Selenium-Binding Protein 1 (SELENBP1) as Biomarker for Adverse Clinical Outcome After Traumatic Spinal Cord Injury
Source: Front Neurosci. 2021 May 28;15:680240. doi: 10.3389/fnins.2021.680240 (PMC8204909; doi:10.3389/fnins.2021.680240)
Supplement: Supplementary file 1 [file Table_1.DOCX]

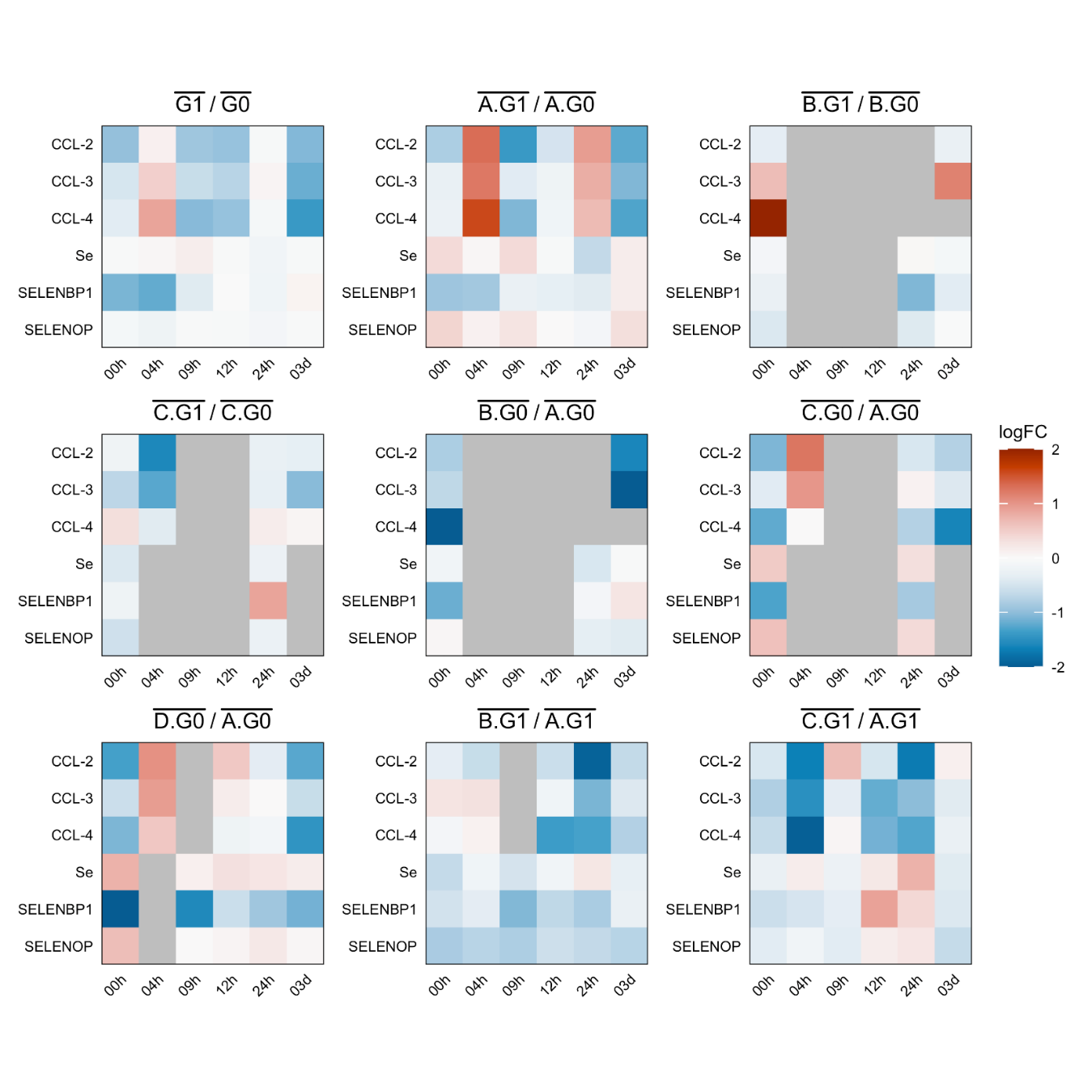


**Suppl. Fig. 1.** Trace element and cytokine dynamics. Heatmap values correspond to cytokine, trace element levels and associated protein concentrations averaged across replicates for each point in time, and z-normalized within each panel (comparison of AIS groups); missing values are shown in grey. Rows correspond to the analytes; columns correspond to 6 points in time from admission (00h) to 3 days after (03d) the injury.
